# Impurities in Glycerin

**Published:** 1868

**Authors:** Henry Bower

**Affiliations:** Philadelphia


### IMPURITIES IN GLYCERIN.

*To the Editor of the Chemical News:*

SIR—The writer of the article on glycerin in *Kunst and Geweberblatt* is correct in attributing the acrid, irritating properties of some glycerin to the mode of preparation; but I have distilled glycerin which was quite as unsuitable for medicinal or surgical purposes as any spoken of. The volatile fatty acids and ethers which exist in crude glycerin are sometimes condensed with the glycerin, and these have very irritating properties.

In the glycerin which is made without distillation, the volatile acids and ethers exist, but not in the same state as after distillation, the high heat required for this process decomposing them into some modification of their original state.

The great cause of irritation in glycerin whieh has not becn properly prepared is the presence of oxalic acid and of formic acid; these are produced by the action of sulphuric acid upon the glycerin, forming the first mentioned acid, and this, in its turn, acts upon the glycerin, giving rise to formic acid, the irritating properties of which are well known.

The nitraie of silver test I have always considered the most reliable. Glycerin, which shows no reaction with this salt, may be considered suitable for all uses, as it indicates not only the presence of chlorine or chlorides, but is, as well, reduced by acids which may exist in the glycerin.

I am, &c., HENRY BOWER.

Philadelphia, Jenuary 16, 1868.

[*Chemical News.*

